# Inflamed Dentine

**Published:** 1857-06

**Authors:** Henry A. Smith

**Affiliations:** Demonstrator of Operative and Mechanical Dentistry in the Ohio College of Dental Surgery


### INFLAMED DENTINE.

BY HENRY A. SMITH, D. D. S.,

DEMONSTRATOR OF OPERATIVE AND MECHANICAL DENTISTRY IN THE OHIO COLLEGE OF DENTAL SURGERY.

There is no pathological condition of the teeth met with more frequently by the Dentist, than that of an inflamed or sensitive condition of dentine, and none which gives him so much trouble and perplexity as this. The patient too, generally suffers in proportion with the perplexity of the Dentist, when operations are performed, where this condition exists. Many operations are but imperfectly performed and the teeth ultimately lost, because of the intense pain caused by a proper preparation of the cavity, previous to filling.

In young persons especially is this the case.

It is important, then, in order to treat this diseased condition successfully, that we should endeavor to ascertain the causes producing it; for success in the treatment of any disease, depends not only upon a knowledge of the structure and functions, but also a knowledge of the causes producing the change of function and structure of the parts.

It is now generally admitted, we believe, the teeth are endowed with all the essential characteristics of vitality. That they have a system of circulation throughout the entire dental tissue, through which it receives nutrition from the general circulation. That this tissue is supplied with nervous fibrils, causing it to become irritable and subject to a grade of inflammatory action, and that it is supplied with absorbents by which it admits of interstitial absorption and deposition to a slight extent.

The term inflammation as applied to the pathological condition we have mentioned, has been objected to by some writers, because all the phenomena of inflammation in other tissues are not present, or at least, are not perceptible.

Redness, heat, pain and swelling, are the four signs distinctive of inflammation. But it is not essential that these should all exist to have inflammation.

In inflammation of dentine we always have pain, and may have redness and heat, although the latter may not be perceptible; but, owing to the dense structure of dentine, never swelling.

Dentine does not ordinarily circulate red blood like the more vascular tissues; but that a nutritious fluid of some kind is carried into it, all admit. A capillary circulation is not essential to the nutrition of a part; for in the most vascular tissues there are portions ("islets in the midst of the capillary net work of vessels,") that must be supplied with nutritive material by absorption by the cells or elementary substance of the tissue, they in turn imparting it to others more distant. This could not extend through any large extent of tissue without lessening the sensibility very much. In this way a portion of fractured bone which is cut off from a supply of blood from the medullary canal, may be supplied with nutrition by absorption, not sufficiently to support vigorous nutrition, but enough to prevent the death of the bone thus removed. The dentine has too great a supply of nerve fibrils, and manifests too great sensibility, especially in parts most distant from the pulp cavity to entertain the theory that it is nourished in this way—an active circulation being necessary to the sensibility of any part supplied with nerves.

The dentine, we think, is supplied with vessels throughout its entire structure, by and through which it receives nutrition. As we have before stated, they do not ordinarily circulate red blood, yet it is not impossible for the red corpuscles to pass into them as is evidenced by the reddened condition of dentine observed. It is objected there is too great difference in size, between the red corpuscles and dental tubuli to admit them. The average diameter of the tubuli is stated to be about 1-10,000 of an inch. That of the red blood disk in man, about 1-3,200 of an inch. But the size of corpuscles is liable to great variation in the same individual—some of them being one third smaller than the average. Their size also often undergoes a change during their circulation, in consequence of pressure, through a narrowing of the channel, becoming elongated, twisted or bent. This change may be so great as to allow the disk to pass through an opening much smaller than its average diameter. In inflamed dentine we may have a reddened condition of the teeth, caused by an aggregation of the red corpuscles within the vessels. The force necessary to drive into the tubuli resulting from a determination to the part, with the motion increased. The red corpuscles may be ruptured, not by "mechanical violence offered to the teeth," but resulting from an altered condition of the blood. A reddened condition of the dentine cannot, we think, be attributed to this cause, nor to an acute inflammation of the pulp alone.

Pain is another of the symptoms mentioned, it always being present. Not only is it a symptom, but the chief element of the disease, and against which remedies must be directed. This pain or increased sensibility is generally the result of chemical agents, acting as an irritant upon the tooth bone, or constitutional derangement, or from both of these causes combined. Caries, the result of chemical agents acting on the tooth bone, acts as an irritant, causing a determination to the part—exalted sensibilty and inflammation resulting.

Any change from a normal condition of the blood may become a predisposing cause to this diseased condition; for the healthy condition of a part depends especially upon a due supply of well elaborated blood, and also upon a normal condition of the part to be nourished.

To every part supplied with nerves, an active circulation is essential. Any cause which retards this deadens the sensibility, and any increase in its circulation, induces an increase in the sensibility of the part.

The mind has a powerful influence in causing a determination to a part, as we perceive in the act of blushing. This is a healthy physiological condition, yet we see no reason why a similar condition may not be produced in a tooth, by having the mind directed to it, causing an increase in its circulation with increased sensibility. Every operator has noticed how patients often shrink and manifest emotions of pain, even before the part is touched with an instrument. In many cases this sensibility is entirely removed after a few cuts with an excavator, or so soon as the patient is convinced his fears will not be realized.

In this way the mind acting as an irritant, may become one of the causes in producing an increase in the sensibility of dentine.

The extent of this inflammation or exalted sensibility varies very materially. We may have it extending throughout the entire body of dentine, or confined to a superficial layer in the cavity—again it is confined to one or more points in a cavity, or at the union of the enamel and dentine. These modifications may be attributed to a variety of causes. They may be owing to the force of the chemical agent acting as an irritant in producing inflammation, or, to the perfect or imperfect development of the dentine. The confinement of it to one or more points may be owing to an imperfect development at those points, causing the agent to act more readily. At the union of the dentine and enamel, we have a greater distribution of nerve fibrils; it being a law of the animal economy that we find the greatest sensibility at the surfaces.

Sensory nerves are distributed generally in the same proportion as blood vessels,—for instance, in the non vascular tissues, as the epidermis, cartilages, nails etc., no vessels exist and there is an absence of sensibility, and in ligaments, tendons and other tissues possessed of no great vascularity, sensibility is dull. In the teeth of young persons we find the greatest sensibility because of a greater vascular circulation. The growing body of the child requires a greater supply of nourishment to sustain it; so the teeth of the young require a greater supply of blood to afford material for their perfect development.

It is maintained by some that this exalted sensibility is not a pathological, but rather a physiological condition. The teeth are supplied with sensory nerves, the nervous system has its function, and any cause producing a change from its natural or healthy performance, may be called a disease of function. If the cause producing this alteration of function, is local, acting as an irritant, the part is restored to health in majority of cases, by a removal of the cause. So long as the cause exists, the pain is persistent, if the vitality of the tooth is not sufficient to overcome the effects of the irritant acting upon it. There is a constant conflict between the chemical and vital forces of the body. In health the vital are able to resist the chemical forces. But if the structure or function of a part become diseased, the chemical forces act with renewed energy. This is the case in inflammation of dentine—the vitality of the tooth not being able to resist the chemical agents acting on the dentine—resulting in a pathological condition. Another evidence that this is a diseased condition as well as inflammation is, that it yields to antiphlogistic treatment. It may not always be necessary to resort to this kind of treatment, unless it be for the purpose of allaying the sensibility that we may remove the carious portion of the tooth. Such remedies as act on the surface only, will generally accomplish this.

The results of this inflamed condition of dentine are not so definite in their nature, nor so easily defined as in many other tissues. The teeth are subject to diseases similar in their nature to those of other osseous structures. But the phenomena presented do not bear so close an analogy—not because of their being very much lower in grade of organization, but in the peculiarity of their situation. The entire crown being exposed to the many external agents causing disease. Caries—that condition most analogous to disease of bones—is the result of external agents, and this being the exciting cause of inflammation in dentine, it is not strange we do not find all the results of inflammation in other and similar tissues, and especially if we remember that the teeth do not possess that recuperative power to any extent that the bones do.

Inflamed dentine generally *resolves* itself into a healthy condition when the local exciting cause is removed and the part shielded. It may proceed until the pulp is involved and the death of the tooth results, or the vitality of it, so far as it is dependent on the pulp for a supply of nutrition. This, however, rarely occurs. When caries has extended to the pulp cavity, the inflammation usually subsides and we do not find much sensibility. This is owing to the healthy function of the nerve not being performed, failing to supply that amount of fluid for a vigorous circulation, necessary in a sensitive condition of dentine.

Induration or hardening is a frequent *result* of this condition—caused by a deposition of osseous material within the vessels; it being carried in by the increased vascular circulation. Whether we have *effusion* as a result of this inflammation is doubtful. Decay of teeth presents a variety of appearances. The amount of sensibility depending somewhat upon the appearance of this decayed portion. Now we may possibly have an effusion altering the character and appearnce of decay and also modifying the sensibility.

If we had all the results of inflammation in similar tissues, presented in this condition, the introduction of gold and other metals to arrest decay, would be very improper, tending to increase the difficulty rather than relieve it. This treatment is based on the theory, that the dentine does not possess that power to restore itself by throwing out granulations like other vascular tissues do, when inflamed. We have, then, as proof that this is an inflamed, as well as a diseased condition, the vascularity of the dental tissue. And any part possessing vascularity may be subject to a grade of inflammation, the usual signs of inflammation or enough to indicate it, viz: pain; and may have heat and redness, and also the fact that it yields to antiphlogistic treatment. The results, if not the same, are to a certain extent similar to those of inflammation in other bony tissues. And finally, that most conclusive of all arguments—if it is not inflammation, what is it?

